# Plasticity of Persistent Activity and Its Constraints

**DOI:** 10.3389/fncir.2020.00015

**Published:** 2020-05-07

**Authors:** Sihai Li, Xin Zhou, Christos Constantinidis, Xue-Lian Qi

**Affiliations:** ^1^Department of Neurobiology and Anatomy, Wake Forest School of Medicine, Winston Salem, NC, United States; ^2^Department of Computer Science, Stanford University, Stanford, CA, United States

**Keywords:** working memory, prefrontal cortex, training, monkey, neurophysiology

## Abstract

Stimulus information is maintained in working memory by action potentials that persist after the stimulus is no longer physically present. The prefrontal cortex is a critical brain area that maintains such persistent activity due to an intrinsic network with unique synaptic connectivity, NMDA receptors, and interneuron types. Persistent activity can be highly plastic depending on task demands but it also appears in naïve subjects, not trained or required to perform a task at all. Here, we review what aspects of persistent activity remain constant and what factors can modify it, focusing primarily on neurophysiological results from non-human primate studies. Changes in persistent activity are constrained by anatomical location, with more ventral and more anterior prefrontal areas exhibiting the greatest capacity for plasticity, as opposed to posterior and dorsal areas, which change relatively little with training. Learning to perform a cognitive task for the first time, further practicing the task, and switching between learned tasks can modify persistent activity. The ability of the prefrontal cortex to generate persistent activity also depends on age, with changes noted between adolescence, adulthood, and old age. Mean firing rates, variability and correlation of persistent discharges, but also time-varying firing rate dynamics are altered by these factors. Plastic changes in the strength of intrinsic network connections can be revealed by the analysis of synchronous spiking between neurons. These results are essential for understanding how the prefrontal cortex mediates working memory and intelligent behavior.

## Introduction

Working memory, the ability to maintain and manipulate information in mind over seconds, is one of the key components of higher cognitive functions (Baddeley, 2012). Early neurophysiological studies identified neurons in the lateral prefrontal cortex that generate persistent activity during working memory tasks (Fuster and Alexander, 1971; Kubota and Niki, 1971). Furthermore, the activity of individual prefrontal neurons was shown to be sensitive to the identity and location of remembered stimuli (Fuster and Alexander, 1971; Funahashi et al., 1989; Constantinidis et al., 2001b), as well as task variables, quantities, and categorical judgments (Freedman et al., 2001; Crowe et al., 2013; Blackman et al., 2016). As a result, information about all of these variables can be decoded from the activity of ensembles of prefrontal neurons (Meyers et al., 2008, 2012). Working memory is not the only cognitive domain that persistent neural activity seems to predict (Constantinidis and Luna, 2019). For example, activity elicited during the preparatory period of an antisaccade task is correlated with the levels of working memory activity, on a neuron by neuron basis (Zhou et al., 2016a). Response preparation is a critical parameter of inhibitory control (DeSouza et al., 2003; Ordaz et al., 2010) and baseline activity may thus be tied to working memory, encoding advance preparation for the upcoming requirement to resist the stimulus appearance.

In recent years, alternative models have been proposed for working memory that do not rely on persistent activity, such as ones that rely on short-term modification of synaptic properties to maintain information, instead (Stokes, 2015; Mi et al., 2017; Lundqvist et al., 2018). It has also been suggested that the rhythmicity of activity generated during working memory is the critical neural variable for maintenance rather than the rate of persistent discharges. The magnitude, frequency and the phase of neural oscillations have indeed been demonstrated to be modulated as a function of stimuli and task information (Lundqvist et al., 2016, 2018). While more than one mechanism may play a role in the representation of information in working memory, these findings do not contradict the storage of working memory information in persistent neural activity (Riley and Constantinidis, 2016; Constantinidis et al., 2018). Modeling studies in which changes in synaptic plasticity are sufficient to maintain information in working memory in some tasks also reveal that persistent discharges are necessary for other, more complex tasks (Bouchacourt and Buschman, 2019; Masse et al., 2019). Only measures of persistent activity are strongly predictive of behavior in working memory tasks (Constantinidis et al., 2001b; Wimmer et al., 2014). We therefore focus exclusively on persistent activity in this review.

Although persistent activity maintains stimulus representations, it is also subject to change, which appears as a result of learning and development. Such plasticity is necessary for and provides a foundation for intelligent behavior. In recent years, neurophysiological and imaging studies have provided new insights into the effects of training in working memory tasks on the prefrontal cortex (Qi and Constantinidis, 2013; Constantinidis and Klingberg, 2016). Human and animal studies have made it possible to investigate how the prefrontal cortex responds to visual stimuli before and after behavioral training in a cognitive task, and how new information is integrated into neural circuits that are simultaneously maintaining information about the stimuli (Olesen et al., 2004; Meyer et al., 2011). Plasticity also occurs at different life stages, for example in adolescence, when the improvement of behavioral performance is associated with changes in prefrontal cortical activity (Constantinidis and Luna, 2019).

To understand the mechanisms of plasticity related to working memory it is necessary to first consider the neural circuits that generate persistent activity. Neural activity is thought to be sustained by reverberations of discharges in a network of neurons with reciprocal and recurrent connections (Wang, 2001; Wimmer et al., 2014; Riley and Constantinidis, 2016; Zylberberg and Strowbridge, 2017). The past decade has seen significant gains in our understanding of how persistent neural activity may change over time. In the current review, we aim to examine the latest insights on this topic. We focus mainly on visual-spatial working memory, the ability to maintain the spatial location of visual stimuli in mind, as this model provides us with a parametric variable, whose representation in neural activity is well understood (Riley and Constantinidis, 2016). We also focus on the lateral prefrontal cortex, the brain region most intricately implicated in this function, in non-human primates (Constantinidis and Procyk, 2004). The following sections review the mechanisms and circuits of persistent activity generation, how and to what extent these are plastic, and the open questions in the field, to be addressed in future studies.

## Mechanisms and Models of Persistent Activity Generation

### Intrinsic Circuits

**Figure 1:**
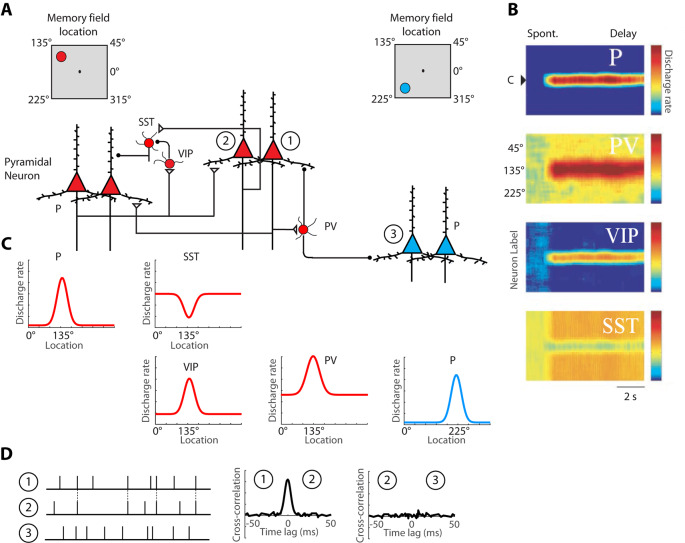
Schematic illustration of the basic intrinsic circuit that maintains persistent activity in the prefrontal cortex. **(A)** Different types of neurons are indicated as follows. P, Pyramidal Neuron; PV, Parvalbumin Interneuron; VIP, Vasoactive Intestinal Polypeptide expressing Interneuron; SST, Somatostatin expressing Interneuron. Open triangles denote excitatory synapses; black circles indicate inhibitory synapses. Insets on top are meant to illustrate that red-colored neurons on the left side of the figure are driven by a stimulus at the upper left of the screen, the 135° location, whereas blue-colored neurons on the right side of the figure are maximally activated by a stimulus in the lower left, 225° location. Excitatory synapses connect pyramidal neurons with similar preferences in the delay period that follows a stimulus in the upper left. **(B)** Heat maps representing the activity of different neurons are plotted by a preference for stimulus location (y-axis), as a function of time (x-axis). **(C)** Tuning curves of the same neuronal population, during the delay period. **(D)** Schematic illustration of cross-correlation analysis for neurons 1, 2, and 3, indicated in panel **(A)**. Raster plots represent spike time series of each neuron, obtained during a baseline period, before the appearance of stimuli. Synchronous spikes between neurons 1 and 2 result in a cross-correlation peak, centered at 0-lag. Adapted with permission from Zhou et al. (2012) and Wang et al. (2004) Copyright 2004 National Academy of Sciences.

Persistent activity depends simultaneously upon the properties of single neurons, the properties of neural networks within a cortical area, and the properties of long-distance networks between cortical areas. The influence of intrinsic prefrontal networks (schematically illustrated in Figures 1A–C) on neuronal activity can be investigated by physiological means. Nearby cortical neurons tend to generate near-synchronous spikes, within 0–2 ms of each other, significantly more often than would be expected by chance (Constantinidis et al., 2001a; Zick et al., 2018). These neurons also tend to be positively correlated at slower time scales, as evidenced by discharge rates averaged over periods in the order of 0.5–1 s (Constantinidis et al., 2001a; Kiani et al., 2015; Leavitt et al., 2017b). Cross-correlation analysis (Figure 1D), quantifying the relative timing of spiking of two neurons at the millisecond scale reveals that, when present, millisecond-scale cross-correlation peaks are most often centered at time 0, indicating synchronous firing (Constantinidis and Goldman-Rakic, 2002; Zhou et al., 2014). This pattern of cross-correlation peak is consistent with two neurons receiving input from common synaptic sources and provides a measure of the strength of intrinsic connections. The degree of synchronization is higher for neurons with similar spatial tuning and neurons active in the same epochs of the behavioral task, as would be predicted for neurons receiving shared input, which results in similar functional properties (Constantinidis et al., 2001a). We rely on cross-correlation measures to make inferences on circuit organization, and plasticity, below.

#### Axonal Projections

Reverberating activity through layer II/III horizontal excitatory connections between neurons with similar stimulus tuning is currently believed to be the primary mechanism of persistent discharge generation (Constantinidis and Wang, 2004). The basic circuit is illustrated in Figure 1A. Anatomical studies identified that prefrontal neurons receive horizontal connections from clusters of cells arranged in 0.2–0.8 mm wide stripes of the cortex, providing an anatomical substrate for such reverberation (Goldman-Rakic, 1984; Levitt et al., 1993; Kritzer and Goldman-Rakic, 1995; Pucak et al., 1996).

Intrinsic connectivity is quantitatively enhanced within the prefrontal cortex compared to other cortical areas. Prefrontal pyramidal neurons exhibit the most extensive dendritic trees and the largest number of spines among cortical neurons (Elston, 2000, 2003). Physiological signatures of this greater extent of synaptic inputs into prefrontal neurons have been found in comparative cross-correlation studies, contrasting different cortical areas. Prefrontal neurons appear to receive a greater percentage of their inputs from neurons located at greater distances (>1 mm), and consequently to share a greater proportion of their inputs with neurons located at longer distances; in contrast, the spatial spread of inputs to posterior parietal neurons is much more limited and neurons located at shorter distances of each other (in the order of 0.2–0.5 mm) share a greater proportion of their inputs (Katsuki et al., 2014).

Other systematic differences between cortical areas in terms of axonal projections have also been identified recently, such as the MRI-based T1-weighted/T2-weighted ratio (Burt et al., 2018). This ratio is indicative of the extent of myelin presence within gray matter and provides a measure of convergence of axonal projections (Glasser and Van Essen, 2011; Huntenburg et al., 2017). This ratio is highest in the primary visual cortex and lowest (indicating most sparse connections) in the prefrontal cortex (Burt et al., 2018).

#### NMDA Receptors

NMDA receptors are critical in any neural circuit that generates persistent activity (Constantinidis and Wang, 2004). The relatively slow decay time constant of NMDA receptor-mediated synaptic currents allows post-synaptic neurons to remain in a depolarized state for a longer time (Wang, 2001). If a network of excitatory neurons contained only AMPA synaptic receptors, which produce synaptic currents with very fast decay time constant, unrealistically high firing rates would be necessary to sustain neural activity during the delay period of a memory task (Wang, 1999). Experimental results also support the role of NMDA receptors in the generation of persistent activity, as NMDA antagonists greatly degrade persistent activity (Wang et al., 2013; Wang and Arnsten, 2015). For example, systemic administration of ketamine, a non-specific NMDA antagonist, decreases the strength of effective connectivity between prefrontal neurons, as evidenced by a decrease in the synchronous spiking between simultaneously recorded neurons (Zick et al., 2018).

NMDA expression is also area-specific. Among the different subunits that compose NMDARs in the adult brain, GluN2B has the slowest decay time constant. A gradient of GluN2B expression exists in the primate brain, with highest levels of expression observed in the prefrontal cortex (Burt et al., 2018), consistent with the ideas originally proposed by Wang (1999) and Wang (2001), that the slow decay constant of synaptic NMDARs is important in models of persistent activity.

Finally, NMDA represents one of the main mechanisms through which dopamine affects persistent activity. Iontophoretic application of dopamine agonists onto prefrontal neurons active during working memory affects firing rate in an inverted U fashion; at moderate doses, they increase activity for preferred stimuli and suppress non-preferred responses (Vijayraghavan et al., 2007; Ott et al., 2014). These agonists enhance the representation of actively remembered stimuli and suppress distractors (Jacob et al., 2016). Computational and experimental studies suggest that dopamine improves the signal-to-noise ratio of persistent activity mainly *via* enhancement of NMDAR currents (Yang and Seamans, 1996; Durstewitz et al., 2000; Seamans et al., 2001; Chen et al., 2004).

#### Interneuron Specialization

Inhibitory neurons in the prefrontal cortex exhibit persistent activity as pyramidal neurons do (Rao et al., 1999, 2000; Constantinidis and Goldman-Rakic, 2002; Constantinidis et al., 2002). Computational models suggest that inhibition is essential for creating stimulus-selective persistent activity (Compte et al., 2000), and both computational and experimental results suggest that prefrontal interneurons generally exhibit higher baseline firing rates and broader tuning than pyramidal neurons (Constantinidis and Goldman-Rakic, 2002).

A division of labor among cortical interneurons has been hypothesized, in which multiple types of GABAergic neurons form a specialized network, to facilitate stimulus-specific persistent activity (Wang et al., 2004), as illustrated in Figure 1A. In this scheme, pyramidal neurons would recruit Parvalbumin (PV) expressing inhibitory interneurons to suppress the activation of other pyramidal neurons, with different spatial turning, since PV cells target the cell bodies of pyramidal neurons. Anatomical evidence that suggests that PFC neurons with similar memory fields are grouped in clusters that may be the anatomical substrate for recurrent excitation (Goldman-Rakic, 1984; Levitt et al., 1993; Kritzer and Goldman-Rakic, 1995; Pucak et al., 1996) and in such a scheme, PV interneurons could provide lateral inhibition by inhibiting neurons in different clusters, as depicted in the model. Alternatively, PV cells may provide feedback inhibition to adjacent pyramidal cells that reciprocally excite the PV cells, as has been demonstrated experimentally in the rodent cortex (Adesnik et al., 2012; Atallah et al., 2012; Wilson et al., 2012). Primate interneurons exhibit broader tuning curves than pyramidal neurons (Constantinidis and Goldman-Rakic, 2002) and in such as scheme, PV neurons would facilitate stimulus-specific working memory by sharpening the tuning function of adjacent pyramidal neurons and contributing to Excitatory/Inhibitory (E/I) balance. Without feedback inhibition, recurrent excitation may shift the E/I balance and bring the network into an unstable, hyper-excited state, which would also be deleterious for the maintenance of working memory (Constantinidis and Wang, 2004).

The second class of inhibitory interneurons, expressing Vasoactive Intestinal Peptide (VIP), 80% of which also express Calretinin (Gabbott and Bacon, 1997), would inhibit a third class of interneurons, those expressing Somatostatin (SST) and likely Calbindin. VIP neurons are interneuron-targeting cells and when activated, they would inhibit SST neurons, which are peridendritic-targeting cells and they tonically inhibit pyramidal neurons (Pi et al., 2013; Dienel and Lewis, 2019). The model predicts that SST neurons exhibit a high spontaneous rate (Figures 1B,C), which during the baseline period, before a stimulus appearance, inhibits tonically all pyramidal neurons. The properties of SST inputs have not been investigated in detail in the primate cortex, but in the rodent cortex, SST neurons are strongly modulated by acetylcholine (Chen et al., 2015; Urban-Ciecko et al., 2018). After a stimulus is maintained in working memory, SST neurons would effectively release from inhibition pyramidal neurons that have already attained a state of excitation by the same stimulus. Other populations of SST neurons, not recruited by the stimulus held in memory would continue to inhibit non-activated pyramidal neurons, thus suppressing background noise as well as potential activation by subsequent, distracting stimuli (Wang et al., 2004).

The activation profiles of these three classes of interneurons and tuning curves relative to the tuning of pyramidal neurons they are linked to are schematically depicted in Figures 1B,C. Direct experimental evidence for the disinhibitory role of VIP cells has been provided by rodent studies (Pi et al., 2013). The model is simplified, in that VIP neurons also inhibit PV neurons, at least in rodent visual cortex. VIP-to-SST and VIP-PV synapses also show strong short-term synaptic depression, which suggests that synaptic output from VIP neurons is best fit to briefly inhibit other interneurons, possibly suppressing the phasic effect of distracting stimuli, rather than being a continuous input during the entire delay period (Pi et al., 2013). Finally, VIP neurons in the mouse barrel cortex are not well-tuned to stimulus properties, suggesting distant inputs (Yu et al., 2019).

Nonetheless, the basic circuit of Figure 1 appears to be conserved in primates. A subset of primate Calretinin interneurons preferentially targets Calbindin interneurons (Meskenaite, 1997; Melchitzky and Lewis, 2008; Fish et al., 2018), thus creating an analogous circuit. Furthermore, interneuron-targeting cells are more abundant in association cortices, and particularly in the prefrontal cortex, compared to the sensory cortex (Defelipe et al., 1999; Elston and González-Albo, 2003). At least indirect evidence supports the idea that a disinhibiting circuit is more pronounced in the prefrontal cortex: interneurons with high baseline firing rate and inverted tuning (consistent with the profile of disinhibiting neurons) are more numerous in the prefrontal cortex than in the posterior parietal cortex (Zhou et al., 2012). While the basic circuit of Figure 1A appears to be present across species and cortical areas, the intrinsic prefrontal circuit is more capable of generating and sustaining persistent activity than its afferent areas.

### Long-Distance Circuits

Although the prefrontal cortex may be the primary source of persistent activity in working memory, the generation of persistent activity is not exclusive to the prefrontal cortex alone. Neurons exhibiting persistent activity have been identified in several additional brain areas, including the posterior, parietal, and inferior temporal cortex, thalamic nuclei, particularly the mediodorsal nucleus of the thalamus, and also the basal ganglia (Constantinidis and Procyk, 2004). This is not to say that persistent activity is entirely distributed across areas, either; it was found to be absent in visual cortical area MT and to emerge *de novo* in area MST, in one well-studied paradigm (Mendoza-Halliday et al., 2014). Long-distance connections between these areas and the prefrontal cortex have been hypothesized to provide a larger scale circuit to generate persistent activity during working memory. Recent modeling efforts suggest that long-range inter-area reverberation may support the emergence of persistent activity in areas whose local circuit organization is not sufficient for its maintenance (Mejias and Wang, 2019). Direct evidence for the necessity of thalamocortical connections for the generation of persistent activity has been provided by rodent studies (Guo et al., 2017). Ultimately, this means that long-distance connections may be essential for the generation of persistent activity both within and outside of the prefrontal cortex.

The existence of persistent activity in multiple brain areas does not necessarily mean that all aspects of working memory are distributed, either (Leavitt et al., 2017a). Instead, different areas appear to be involved with during aspects of working memory (Riley and Constantinidis, 2016). The prefrontal cortex is uniquely equipped to represent information about the spatial location of an initial stimulus after distracting information has been presented, whereas the posterior parietal cortex seems to track the most recent stimulus (Qi et al., 2010). Neuronal activity related to executive control of information maintained in memory is similarly thought to originate in the prefrontal cortex and be transmitted to the posterior parietal cortex (Crowe et al., 2013). The distinction between the patterns of activity in the posterior parietal and prefrontal cortex, however, depends on the parameters of the specific working memory task that is being performed. Under some tasks, the posterior parietal and prefrontal cortex may represent different types of information, encoding either the initial or subsequent stimuli (Jacob and Nieder, 2014; Qi et al., 2015; Masse et al., 2017).

### Linking Circuit Models With Behavior

Persistent activity recorded in the prefrontal cortex is predictive of behavior in working memory tasks. Trials in which the preferred stimulus of a recorded neuron elicits less activity than average are more likely to result in errors (Funahashi et al., 1989; Zhou et al., 2013). As a result, a near-linear relationship between behavioral performance and persistent neural activity has been revealed in tasks that parametrically modulate the properties of stimuli held in working memory (Constantinidis et al., 2001b). Choice probability analysis, comparing the distributions of firing rates in the delay period of correct and error trials, also reveals a stronger relationship between persistent activity in the prefrontal cortex and behavioral outcomes, compared to other areas (Mendoza-Halliday et al., 2014).

Computational models provide a link between persistent activity and behavioral performance in working memory tasks. Persistent activity is sustained in these models by recurrent connections between neurons with similar tuning for stimulus properties, thus allowing activation to be maintained past the presence of the afferent input (Compte et al., 2000; Murray et al., 2017). The system can be thought of as a continuous attractor. Drifts in neuronal activity across the network of prefrontal neurons predict precisely the relationship between firing rate and the endpoint of the saccade (the spatial location being recalled by the monkey) in the ODR task (Wimmer et al., 2014). For example, persistent activity recorded from trials in which monkeys make eye movements deviating clockwise vs. counterclockwise relative to the true location of the stimulus yields slightly different tuning curves, as would be expected if the location recalled was determined by the peak of activity at the end of the delay period.

## Plasticity

The plasticity of neural activity is essential for intelligent behavior. Persistent activity can be highly plastic and is influenced by several factors that also impact working memory performance. This is not to say however that there are no limits in plasticity. The following sections examine plastic changes and their constraints as a result of training and age (section 3.1), and the circuit changes that likely mediate them (“Cellular Substrates of Plasticity” section).

### Initial Working Memory Training

Persistent activity appears to be generated automatically, in subjects not required or even trained to perform a task (Meyer et al., 2007; Riley et al., 2017). When naïve monkeys are passively viewing stimuli, some prefrontal neurons become activated and continue to discharge after the stimuli are no longer present. Working memory has sometimes been thought to require willful effort, and/or training in specific working memory tasks (Postle, 2006). In our everyday experience, however, we can track our environment and recall information even when not explicitly prompted to do so ahead of time, implying that working memory may be an automatically generated process. In agreement with this intuition, a proportion of prefrontal neurons are persistently active even when the subjects were not assigned to remember any stimuli (Meyer et al., 2007; Riley et al., 2017). Furthermore, the rate of persistent discharges in this population is selective for properties of the stimuli, including spatial location, color, and shape. It therefore appears that a prefrontal circuit is hardwired to automatically generate persistent activity once activated by sensory stimuli. However, there are limits to the information that may be represented automatically. For example, the identity of a stimulus generally did not survive a second stimulus presentation in the experiments discussed above, and information about whether the shape of two stimuli was the same or not was largely absent in naïve animals (Meyer et al., 2007; Meyers et al., 2012; Riley et al., 2017).

Training to perform a working memory task for the first time does elicit plastic changes in persistent activity (Mendoza-Halliday and Martinez-Trujillo, 2017; Riley et al., 2018). More neurons are active after such training and generate a higher level of persistent activity (Meyer et al., 2007; Riley et al., 2017). The circuit changes that training induces appear to be lasting and the difference in firing rate between naïve and trained animals are evident even when the trained monkeys are tested with the passive presentation of stimuli, in the same fashion they did before training. The mean firing rate of persistent discharges is also higher in the trained than that in naïve monkeys, though the execution of the task further amplifies persistent activity compared to the passive viewing of stimuli (Riley et al., 2017). Plasticity is not all-or-none in terms of exposure to training. Increases in firing rate tended to accrue with cumulative training and are reflective of the level of performance in the working memory task at each point in time (Qi et al., 2011; Tang et al., 2019). These effects represent average changes in neuronal activity, sampled from different groups of neurons at different stages of training. It will be interesting for future experiments to track the activity of individual neurons as learning of a new task takes place.

### Anatomical Constraints on Plasticity

**Figure 2:**
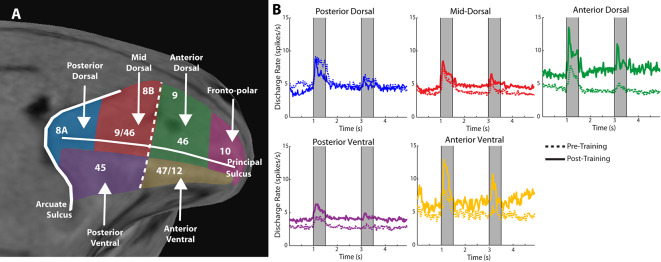
**(A)** Anatomical MRI of the monkey lateral prefrontal cortex with anterior/posterior and dorsal/ventral subdivisions indicated, relative to the Principle and Arcuate Sulci. **(B)** Mean firing rate of neurons recorded in these subdivisions in monkeys both before and after they were trained to perform spatial working memory tasks. Gray bars represent stimulus presentations. Data are shown separately for each prefrontal region. Adapted with permission from Riley et al. (2018).

Anatomical position is an important constraint on the plasticity of persistent activity. Within the lateral prefrontal cortex, levels of persistent activity depend on position across the dorsoventral (Kadohisa et al., 2015; Constantinidis and Qi, 2018) and anterior-posterior axes (Riley et al., 2017, 2018). The lateral aspect of the prefrontal cortex is subdivided into areas 8a, 46, 8b, and 9 in its dorsal aspect, areas 12 and 45 in its ventral aspect, and area 10 covering the frontal pole (Walker, 1940). There is also evidence of a specialization in the anterior-posterior aspect, with the caudal aspect of area 46 shown to be functionally dissociable from the anterior aspect; the former is referred to as area 9/46, whereas the most anterior area is called area 46, in this nomenclature (Petrides, 2000). Division in more areas has also been proposed, based on the evidence provided by fMRI studies probing functional connectivity at rest (Goulas et al., 2017). Based on physiological evidence, we have recently proposed dividing the lateral PFC into subdivisions as follows (Figure 2A): a posterior, mid-, and anterior-dorsal region, a posterior- and anterior-ventral region, and a frontopolar region (Riley et al., 2017).

Neurons in different prefrontal subdivisions exhibit different properties and aptitudes for plasticity (Figure 2B). The posterior aspect of the prefrontal cortex is the most specialized for stimulus location (posterior-dorsal) and object information (posterior-ventral) but is affected relatively little by training (Constantinidis and Qi, 2018). Little difference in mean persistent firing rate is observed in the posterior-dorsal prefrontal cortex before and after training, though more neurons become active (Meyer et al., 2011; Riley et al., 2018). Instead, most of the plasticity in persistent activity occurs in the mid-, and anterior-dorsal areas of the prefrontal cortex (area 46). Across the medio-lateral axis of the dorsal prefrontal cortex, little or no changes in plasticity are seen in the most dorsal areas (areas 8b and 9), whereas plasticity of persistent discharges is evident in the principal sulcus region (area 46), and more so in the ventrolateral prefrontal cortex (Meyer et al., 2011). The organization of the prefrontal cortex has been a matter of debate, with at least some studies failing to identify dissociable responses of neurons in different prefrontal subdivisions (Rao et al., 1997; Lara and Wallis, 2014). In terms of plasticity, however, there seems to be more agreement, and lesion studies support the idea of ventral areas being more essential for the acquisition of new tasks, which implies greater capacity for plasticity (Buckley et al., 2009).

### Plasticity Changes Beyond Firing Rate

Encoding of information in neuronal firing depends not only on the mean firing rate of neuronal responses, but also on how variable these responses are from trial to trial, and on whether firing rates of neurons are positively correlated with each other, which limits how much information can be stored in their collective discharges (Moreno-Bote et al., 2014). The effects of plasticity similarly affect not only mean firing rate but also the variability of persistent activity (Qi and Constantinidis, 2012b) and the correlation of firing rate between simultaneously recorded neurons (Qi and Constantinidis, 2012a). The Fano factor of spike counts, a measure of variability, generally decreases after practicing the task, with the greatest decreases observed in neurons that exhibit persistent activity, compared to neurons that do not. This decrease in trial-to-trial variability may be responsible for increasing the reliability of stimulus property representation after training. Similarly, the spike-count correlation of persistent firing rates between pairs of neurons (known as noise correlation) also decreases after training, which improves the information that can be decoded from simultaneously active neurons (Qi and Constantinidis, 2012a).

**Figure 3:**
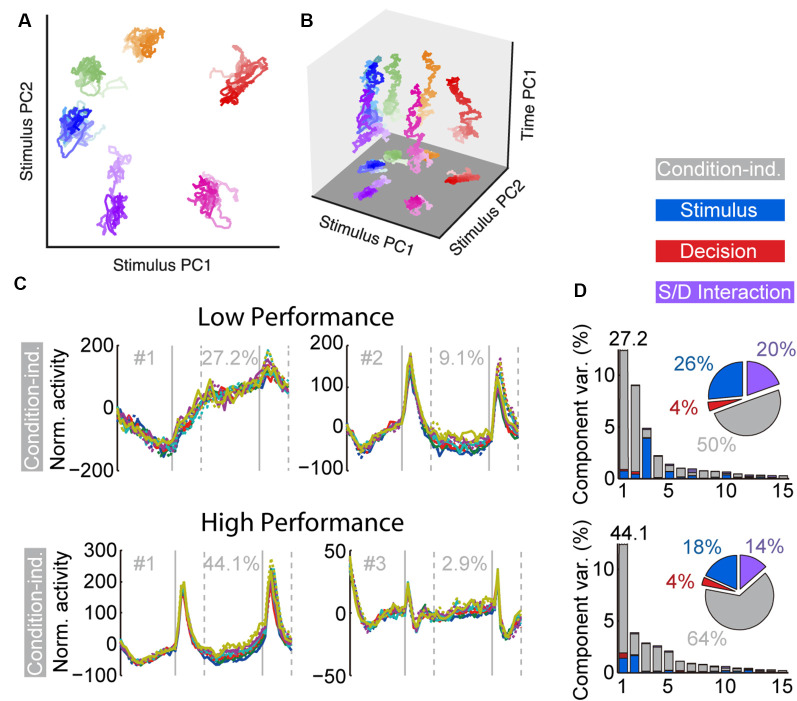
**(A)** Population trajectories during the delay period projected into the mnemonic subspace, defined *via* PCA on time-averaged delay activity. Here the x and y axes display the first and second principal components (PC1 and PC2) of the subspace, respectively. Each trace corresponds to a stimulus condition, roughly corresponding with the actual stimulus location on the screen. The shading of the traces marks the time during the delay, from early (light) to late (dark). **(B)** Same data as in **(A)**, with the z-axis denoting time. **(C)** Demixed PCA Analysis. The first two condition-independent components of dPCA analysis from low and high-performance sessions are plotted, based on an experiment requiring monkeys to maintain multiple stimuli in working memory. **(D)** Histogram representing the amount of variance accounted by different condition-independent, stimulus, decision, and interaction components. Reproduced with permission from Murray et al. (2017; **A,B**) and Tang et al. (2019; **C,D**).

Task training also alters the time course and dynamics of persistent activity (Kobak et al., 2014; Tang et al., 2019). Prefrontal neurons are known to exhibit dynamics during working memory tasks. For example, the firing rate of some neurons is known to “ramp up” or decrease during the trial, so that information about the stimulus is encoded dynamically at different time points (Romo et al., 1999; Meyers et al., 2012; Stokes et al., 2013). However, the existence of dynamics does not undermine the representation of information in working memory. Recent work (Murray et al., 2017) has revealed a stable subspace, where information can be maintained in an invariant fashion (Figures 3A,B). Similar subspaces have been identified across a variety of working memory tasks (Murray et al., 2017; Spaak et al., 2017; Parthasarathy et al., 2019). Training in a working memory task does alter the dynamics of persistent activity. Neuronal responses recorded in animals trained to perform a working memory task exhibit more pronounced increases and decreases of activity during the time course of the trial than animals passively viewing (Kobak et al., 2014; Tang et al., 2019). The consequence of this change is that a greater percentage of firing rate variance is accounted for by components unrelated to the remembered stimulus location or identity.

### Plasticity When Learning to Perform Additional Tasks

After monkeys have been trained to perform basic cognitive tasks, it is possible to train them in more complex tasks, including ones requiring working memory for multiple stimuli. Training in tasks with multiple-stimuli can improve working memory capacity (at least in the task trained) and induces plastic changes in prefrontal activity (Tang et al., 2019): more neurons become activated, their baseline firing rate decreases, and although persistent activity may not change appreciably, the rate of persistent activity relative to baseline is enhanced after training. A debate exists in the human imaging literature, with some studies revealing decreases in activity after training in complex tasks (Schneiders et al., 2011; Kühn et al., 2013; Schweizer et al., 2013; Takeuchi et al., 2013) and the decreases are often interpreted as improvements in efficiency, or strategy (Constantinidis and Klingberg, 2016). The decrease in baseline activity observed in the neurophysiological studies may be partially responsible for such results, particularly when activity is averaged over long periods, as in fMRI studies. Acquiring data during training in a variety of tasks will be essential for understanding the full repertoire of plastic changes.

Like humans, monkeys are known to develop strategies when attempting to master complex tasks, e.g., suggestive of a grouping of multiple stimuli in memory based on their geometric arrangement (Tang et al., 2017). The selectivity of prefrontal responses for remembered displays containing multiple stimuli, or sequences of remembered stimuli, is often very different than for single, identical stimuli (Konecky et al., 2017; Tang et al., 2019) depending on the corresponding mental operation performed.

An important finding of the training studies with multiple stimuli was changed in the dynamics of neuronal activity (Tang et al., 2019). As was the case with the initial working memory training, once subjects practiced a new task requiring memory for multiple stimuli and improved their performance, a greater percentage of activity could be explained by “condition-independent” components, not related to the stimuli being remembered (Figures 3C,D).

Learning to perform multiple tasks also alters the levels of persistent activity representing the newly acquired information (Sarma et al., 2016). Modulation of persistent activity depending on what information needs to be maintained in memory can take place very rapidly, e.g., within a few trials, when a subject learns a new sensory-motor association (Asaad et al., 1998) or on a trial-to-trial basis, when the subject is cued to remember a particular feature of the stimulus to perform judgment and ignore others (Mante et al., 2013). The persistent activity can also be modulated in the course of a single trial during the execution of dual-task paradigms when the subject temporarily focuses on the representation of one stimulus in memory before resuming a task requiring representation of another stimulus (Watanabe and Funahashi, 2014).

### Age

**Figure 4:**
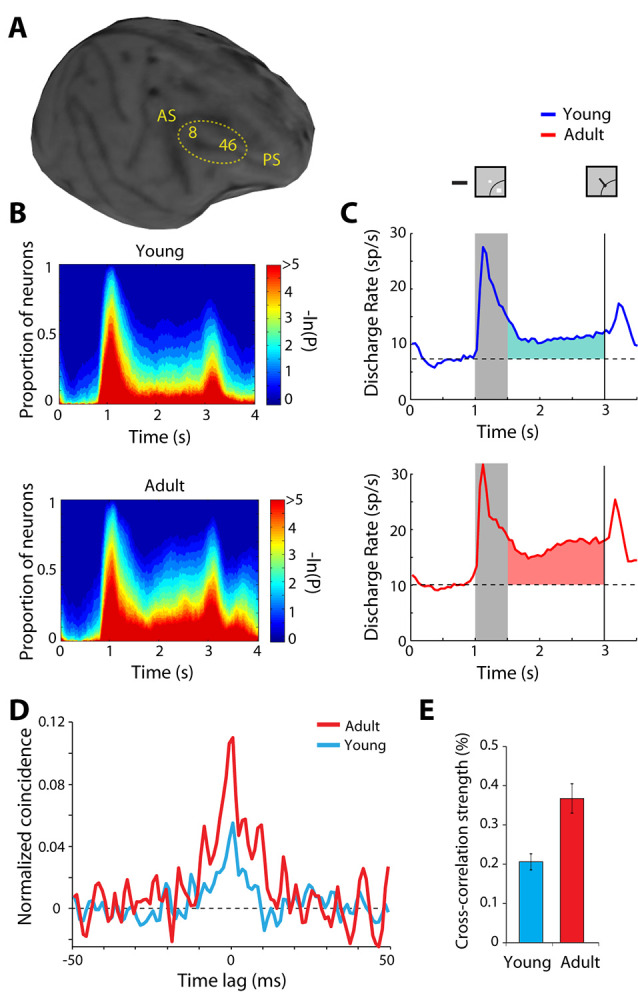
**(A)** Anatomical MRI of an adolescent monkey, with the dorsolateral cortex (areas 8 and 46) indicated. AS, Arcuate Sulcus; PS, Principal Sulcus. **(B)** The proportion of neurons with significant differences in firing rates compared to baseline fixation, at different time points of the ODR task (color scale represents the probability of a paired *t*-test), for the adolescent and adult groups. **(C)** Average population peri-stimulus time histogram for neurons that responded to the visual stimulus and were recorded during the ODR task in the adolescent stage (top) or the adult stage (bottom). Responses are shown for a stimulus in each neuron’s receptive field. Gray bar represents an interval of stimulus presentation; the vertical line represents the time of the fixation offset. Insets schematically display the stimulus location and direction of eye movement relative to the receptive field (arc), which varied for each neuron. **(D)** Averaged, normalized cross-correlation histogram for adolescent and adult monkeys. **(E)** Average value (strength) calculated in the center 5 ms of the cross-correlation histogram for adolescent and adult monkeys. Adapted with permission from Zhou et al. (2014, 2016b).

The normal developmental and aging process provides another opportunity to study plastic changes in the ability of the prefrontal cortex to generate persistent discharges, regardless of training and life experiences. Behavioral performance and neural activity in working memory tasks change markedly around the time of puberty, a developmental event associated with the release of sex hormones and significant neurological change (Zhou et al., 2013, 2016b). The performance of working memory tasks is subtly but significantly higher in adult monkeys compared to adolescent monkeys that have entered puberty, just as it improves in humans between these two developmental stages (Montez et al., 2019). Persistent activity is also higher in adult animals than adolescent ones (Figures 4A–C). Even when comparing persistent activity from adolescent and adult monkeys obtained in sessions equated for performance, the adult prefrontal cortex is better able to generate persistent activity. Furthermore, the adult prefrontal cortex can more effectively filter distracting stimuli during working memory.

In the other end of the life spectrum, advanced age in monkeys is marked by a significant loss of persistent firing in the prefrontal cortex. Aged animals exhibit elevated cyclic-AMP (cAMP) signaling, which reduces persistent activity by opening Hyperpolarization-activated Cyclic Nucleotide–gated channels (HCN—nonselective voltage-gated cation channels), and KCNQ (Potassium voltage-gated channels). The persistent activity can be partially restored to more youthful levels by inhibiting cAMP signaling, or by blocking HCN or KCNQ channels (Wang et al., 2011). Notably, both in the adolescent and aged monkeys it was the persistent activity that differed from that recorded in adults (Wang et al., 2011; Zhou et al., 2016b). Prefrontal activity during the stimulus presentation differed little between age groups, suggesting that stimulus-driven neuronal responses were fully mature in puberty and resistant to the effects of aging in old monkeys. These studies provide some brief snapshots of neuronal activity at two critical life stages. It will be important for future studies to reveal the full-time course of persistent discharges from young to old age.

### Cellular Substrates of Plasticity

The observed changes in neural activity through training and age suggest plasticity in the circuit that generates persistent discharges. Local-circuit differences are evident between adolescent and adult monkeys that could explain the decreased ability of the immature prefrontal cortex to generate persistent discharges. Zero-lag spiking synchronization based on cross-correlation analysis of nearby neurons (recorded at distances between 0.5–1 mm from each other) is markedly lower in adolescent than in adult monkeys (Figures 4D,E). This difference is primarily the effect of changes in inhibitory interactions (Zhou et al., 2014), possibly due to decreases in the connectivity strength of pyramidal neurons onto interneurons, which lessens the net output of inhibitory connections as the prefrontal cortex matures (Gonzalez-Burgos et al., 2015). Interestingly, a decrease in zero-lag synchrony of prefrontal neurons has been recently implicated in schizophrenia (Zick et al., 2018), a condition that, among other pathological symptoms, compromises working memory.

Neuromodulators have also been implicated in dynamic changes of persistent activity and are likely to be involved during learning or the selection of stimulus features. Most notably, cholinergic stimulation through the iontophoretic application of cholinergic agonists (Yang et al., 2013; Sun et al., 2017; Dasilva et al., 2019), or the stimulation of the cholinergic basal forebrain (Qi et al., 2019) leads to a general increase in activity of neurons in the prefrontal cortex. Conversely, systemic administration of the muscarinic antagonist scopolamine (Zhou et al., 2011) or iontophoresis of muscarinic and nicotinic-α7 inhibitors seems to depress prefrontal persistent activity (Yang et al., 2013; Major et al., 2015; Dasilva et al., 2019). Cholinergic stimulation elicits not only direct changes in neural activity but also long-term neuroplasticity effects, suggestive of circuit reorganization (Brzosko et al., 2019).

The reason that plasticity differs between PFC subdivisions can also be traced to systematic differences between anatomical connections and cellular mechanisms. Anatomical studies point to relative segregation of projections from the posterior parietal cortex, which terminate mostly to the posterior dorsal PFC (areas 8 and 46, including both banks of the principal sulcus), and from the inferior temporal cortex, which terminate on the posterior ventral PFC (Petrides and Pandya, 1984; Selemon and Goldman-Rakic, 1988; Cavada and Goldman-Rakic, 1989). Areas higher in the sensory and limbic hierarchies projecting to more anterior prefrontal subdivisions (Gerbella et al., 2013; Barbas, 2015; Borra et al., 2019). Increasingly anterior prefrontal areas integrate inputs from more posterior ones, being activated by higher-order cognitive operations in a rostral-caudal axis of cognitive control (Petrides, 2005).

A greater capacity for plasticity after training in a cognitive task may also point to the specialization of underlying cellular and molecular mechanisms (Kuboshima-Amemori and Sawaguchi, 2007), which may vary between prefrontal subdivisions. Indeed, direct evidence of systematic variation of plasticity markers between limbic and eulaminate areas has been recently documented in the prefrontal cortex (García-Cabezas et al., 2017). Calcium/calmodulin-dependent protein kinase II (CaMKII), which is essential for plasticity, is more impoverished in area 46d compared to more anterior limbic areas, whereas makers of cortical stability, including intracortical myelin, perineuronal nets, and PV show the reverse pattern. Changes in neuronal morphology, molecular profiles of the synaptic apparatus, and the influence of neuromodulator systems have also been implicated in long-term prefrontal plasticity (Laroche et al., 2000; McEwen and Morrison, 2013), and may differ between areas. Finally, short-term synaptic plasticity, depression or facilitation, has been documented in the prefrontal cortex, and this too may be critical, particularly for task-related plasticity (Hempel et al., 2000). Tying these cellular and molecular mechanisms to actual changes in neuronal activity and capacity for plasticity will be an important goal for future studies.

## Conclusions and Open Questions

This review summarized the current state of knowledge on the generation and plasticity of persistent activity during working memory. Some conclusions emerge from this review. We conclude that although the prefrontal cortex is not the only area where persistent discharges are evident, its unique cellular and circuit organization makes it essential for the generation of persistent activity. Training in working memory tasks greatly affects the neuronal circuit of the prefrontal cortex, causing more neurons to exhibit persistent activity and to have this activity reach higher discharge rates. Different prefrontal subdivisions have different capacities for plasticity, with most plastic changes being evident in anterior and ventral areas. Plasticity may manifest itself in a variety of ways. Higher firing rate during the delay period is the most obvious effect of training and the adult stage of maturation, compared to adolescence and old age. However, changes in firing rate variability, correlation between firing rates of different neurons, decreases in baseline firing rate, and changes in neuronal dynamics have all been identified as markers of plasticity.

Many questions related to the generation and plasticity of persistent activity remain open for future research: first, we hypothesized that the functional circuit of Figure 1 is most developed in the primate prefrontal cortex. Testing of the role of identified interneuron populations in different cortical areas in the context of working memory could provide direct evidence that this is the case. Second, what are the actual synaptic changes that occur at the level of intrinsic circuits, within the prefrontal cortex, as well as in long-range connections between the prefrontal cortex and other areas when subjects learn and practice working memory tasks? Addressing this question will require the interrogation of circuits in subjects while they learn to perform working memory tasks. Recent technical developments have brought this aim within reach. Next, what is the role of different neurotransmitter systems during learning? It is well understood that dopamine and acetylcholine play an essential role in neuroplasticity but there is a gap regarding how these factors affect persistent activity during training. A final area of unanswered questions has to do with the generation of object memory. Although spatial memory can be manipulated parametrically and modeled in a neural circuit, object memory has proven more elusive. We therefore ask how objects are maintained in working memory and the factors that govern plasticity for object memory. These questions will have to be addressed in future studies.

## Author Contributions

CC and X-LQ conceived and organized the article. SL, XZ, CC, and X-LQ authored the text jointly.

## Conflict of Interest

The authors declare that the research was conducted in the absence of any commercial or financial relationships that could be construed as a potential conflict of interest.
